# Eight-year Follow-up of Malignant Fibrous Histiocytoma (Undifferentiated High-grade Pleomorphic Sarcoma) of the Maxilla: Case Report and Review of the Literature

**DOI:** 10.5681/joddd.2009.009

**Published:** 2009-03-16

**Authors:** Hossein Shahoon, Mostafa Esmaeili, Mahsa Nematollahi

**Affiliations:** ^1^Assistant Professor and Head, Department of Oral and Maxillofacial Surgery, School of Dentistry, Shahed University of Medical Sciences, Tehran, Iran; ^2^Senior Resident, Department of Oral Medicine and Diagnosis, School of Dentistry, Shahed University of Medical Sciences, Tehran, Iran; ^3^Dentist, Private Practice, Tehran, Iran

**Keywords:** Oral cancer, malignant fibrous histiocytoma, maxilla, undifferentiated high grade pleomorphic sarcoma

## Abstract

Malignant fibrous histiocytoma (MFH) is the most common soft tissue sarcoma of late adult life and may also arise as a primary tumor in bone. It is a rare condition that constitutes less than 1% of the malignant tumors of bone and commonly occurs in the mathaphysis of long bones of extremities such as the femur and tibia. The occurrence in the head and neck region is very rare. MFH of the jaws is a highly malignant tumor that recurs, metastasizes, and usually causes death despite aggressive surgical therapy. We present a case of MFH of maxilla with 8 years follow-up. The clinical, pathologic and radi-ographic features as well as the treatment of this case are discussed.

## Introduction

Malignant fibrous histiocytoma (MFH) is the most common sarcoma of soft tissue in the late adulthood. It may also arise as a primary neoplasm in bone, a rare lesion that constitutes less than 1% of the malignant tumors of bone, commonly occuring in the mathaphysis of long bones of extremities such as the femur and tibia.^1 - 3^ This tumor is very rare in the head and neck region. The mandibulomaxillary area is the most common site for MFH in the skull.^4 , 5^ MFH of the maxilla and mandible is a highly malignant tumor that recurs, metastasizes, and commonly results in death despite aggressive surgical therapy.^6^ The etiology of this tumor is unknown but seems to be multifactorial. Genetic background, environmental factors such as trauma, radiotherapy and malignant transformation from benign lesions can be involved in the etiology of this malignant tumor.^7 - 9^ In this paper, we present a case of MFH of maxilla with eight-year follow-up. The clinical, pathologic and radiographic features as well as the treatment of this case are discussed.

## Case report

**Figure 1:**
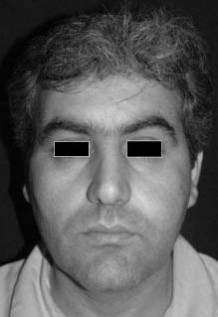
Mild swelling of the left cheek.

A 36-year old man was referred to the Department of Oral and Maxillofacial Surgery, Taleghani Hospital, Tehran, Iran in September 26, 2001 with a mild and painful swelling for two months mostly in the left cheek and palate (Figure 1). The patient’s medical history and a general physical examination revealed no abnormal findings. Regional lymph nodes were not palpable. The history of trauma was negative. A soft movable mass, 3.5 cm in diameter and tender on palpation, was present on the left cheek. There was no numbness of the upper lip.

CT scan of the upper jaw showed an osteolytic mass with extensions to surrounding soft tissues. The margins were ill-defined (Figure 2).


Figure 2. Computed tomography scans showing an extensive osteolytic mass in the left maxilla (a) with extention to the orbital floor and lateral nasal (b).

**a:**
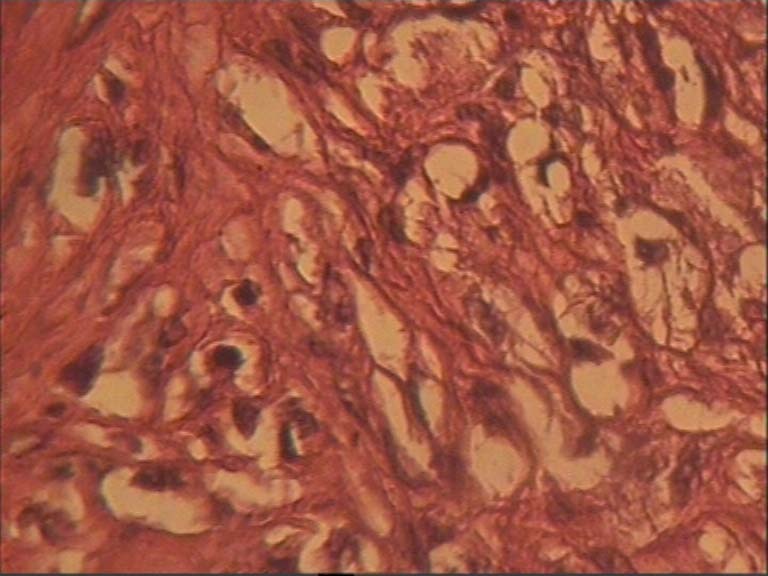

**b:**
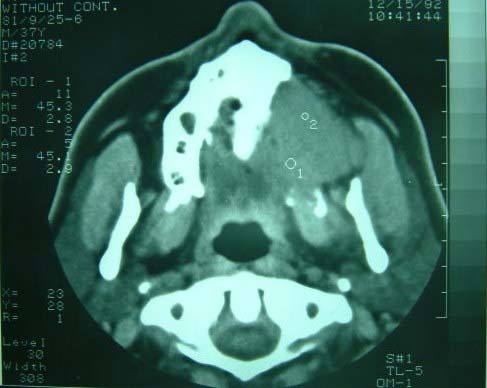

**Figure 3:**
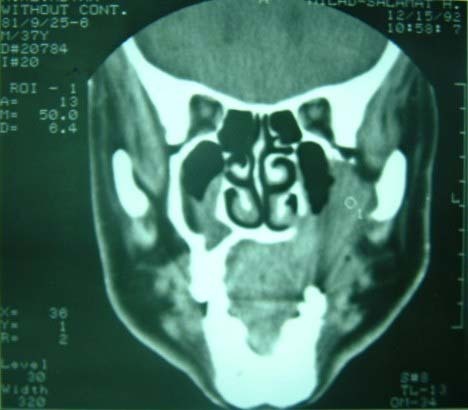
Pleomorphic spindle shaped cells within a myxoid stroma. Intense pleomorphism and multinucleated giant cells exist (hematoxylin and eosin staining, ×100).

An incisional biopsy was performed under local anesthesia. Histological evaluations showed the tumor mainly consisted of spindle cells and atypical multinucleated giant cells. Spindle cells were arranged in a storiform pattern. No other specific features of differentiation were observed in the specimens (Figure 3).

Immuno-histochemical staining revealed positive reactivity for vimentin and α-1 antitrypsin. There was no immuno-reactivity for smooth muscle actin, S100 protein, desmin, MyoD_1_, and cytokeratin. The tumor was diagnosed as high-grade MFH, storiform-pleomorphic type, of the maxilla.

On October 4, 2001, patient underwent surgery. The Weber Ferguson incision and also lip split were performed. Hemi-maxillectomy with orbital floor extension was performed to allow for safety margins.

Frozen sections were performed in almost twenty regions including soft and hard palate, pterygoid muscles, cheek tissue, upper lip, lateral nasal and orbital floor. Evaluation of the sections revealed all areas were free of tumor. A dermal graft from medial thigh region was used to prevent histocontraction. Subsequently, the wound was dressed.

The patient was discharged three days after operation and the sutures were removed eight days later. After surgical treatment, the patient received post operative radiation with 55 Gy during two months utilizing the combination regimen of Adriamycin, dacarbazine and cyclophosphamide. A maxillofacial prosthesis (obturator) was constructed for the patient three months after surgery.

**Figure 4:**
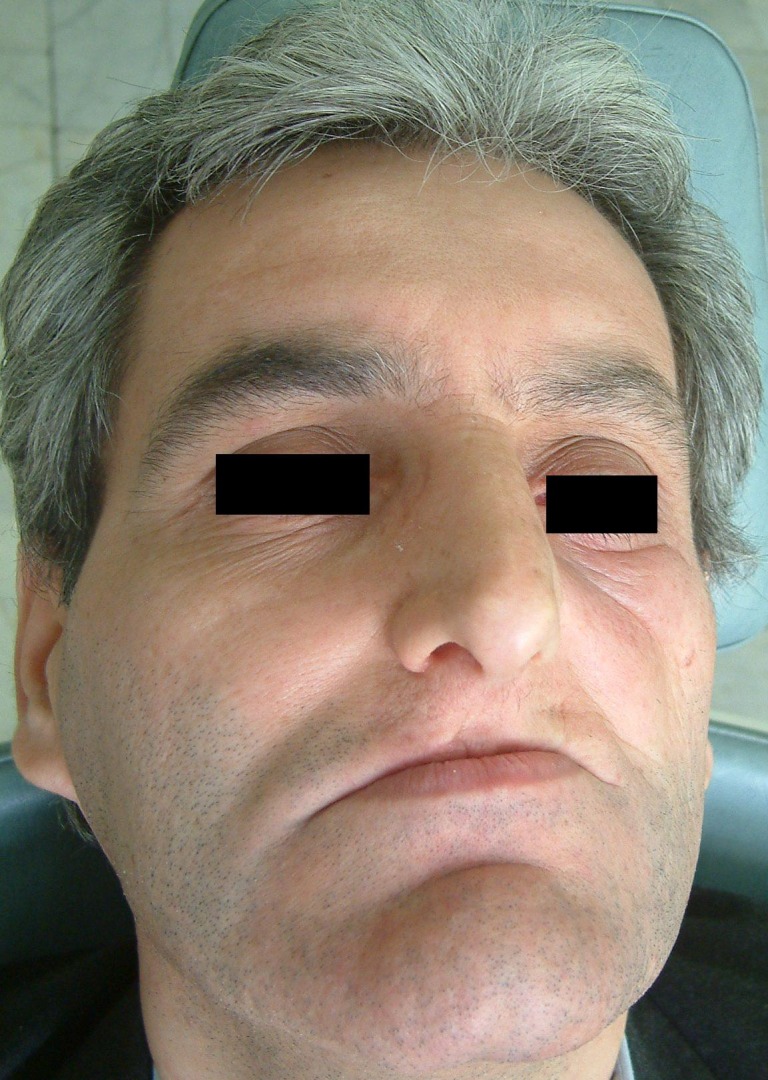
The patient’s frontal view in the 8-year follow-up.

From 2001 to 2009, the patient was visited every year. The patient has been free of local recurrence or distant metastasis of the disease for eight years after surgery (Figure 4).

## Discussion

Malignant fibrous histiocytoma (MFH), the most common soft tissue sarcoma of late adulthood, was first described by O’Brien & Stout in the 1960s.^10^The tumor usually affects individuals older than 60 years of age. Its incidence in young people is less than 5%.^1^ Feldman & Norman^11^ for the first time in the 1970s described primary malignant tumor of bone that satisfied the histopathologic criteria of MFH. MFH of bone constitutes approximately 5% of primary bone tumors and less than 1% of malignant tumors of bone.^1^ This tumor mostly occurs in the mathaphysis of long bones of patients aged 10–60 years, commonly in the distal femur and proximal tibia.^12^ 20% of MFH cases in bone occurs in abnormal bone. MFH is very rare in the head and neck region, with involvement of only 5% to 6% of cases.^1^ The mandibulomaxillary region is the most common area for MFH in the skull.^4 , 5^ MFH of the jaws is a highly malignant tumor that recurs, metastasizes, and commonly results in death despite aggressive surgical therapy.^6^ Most of the patients with MFH of maxilla and mandible show a painful mass or dental problems and the average interval from the first symptom until diagnosis is 3.5 months. Similar features have bean reported for osteosarcoma.^13^

MFH is a neoplasm with fibroblastic nature and facultative histiocytic differentiation. Goldblum et al^14^ described this tumor with four types of histologic presentation, i.e. storiform pleomorphic, mixoid, giant cell, and inflammatory. Many cases of MFH of bone were previously diagnosed as pleomorphic reticulum cell sarcoma, osteolytic fibroblastic osteosarcomas or spindle cell and giant cell sarcoma. MFH of bone is differentiated histologically from osteosarcoma by features such as spindle shape fibroblastic cells in a storiform pattern without osteoid formation. It is usually difficult to differentiate MFH from other malignant tumors of bone including osteosarcoma, leiomyosarcoma, fibrosarcoma, dedifferentiated chondrosarcoma and lymphoma. ^1 , 2 , 13^

The diagnosis of MFH is based on positive immunostaining with vimentin, α1-antitrypsin, and α1-antichymotrypsin.^13 , 15 , 16^

Signs and symptoms of the MFH in head and neck region are similar among different cases which consist of a painful swelling that may be tender on palpation, similar to the present case.^1^ In one case, paresthesia of the lower lip is reported.^7^

The etiology of this tumor is unknown and seems to be multifactorial.^9^ In the present case, according to the patient’s history, the only relative point was presence of two cases of breast cancer in his family. We did not find any obvious inducing environmental factor or any history of systemic disease. Therefore, the genetic background seems to be the most important point in the etiology of this case.

The prognosis of this tumor is often unfavorable and recurrence rate is approximately 44–48%. Metastases more commonly occur in the lung (90% of metastasis) followed by lymph nodes (12%), bone (8%) and liver (1%). About 5% of cases already reveal metastasis when the primary tumor is diagnosed.^1^MFH of jaws commonly result in death despite aggressive therapy.^6^ One of the most important points of this case is the survival of the patient free of local recurrence and distant metastasis, without involving the curettage of the eye in the treatment, which is a very rare instance. In most of the previous cases, the patient has died despite surgical excisions with wide margins.^9^ Therefore, the prognosis of this case seems to be better than most similar cases and the reason may be the early diagnosis and surgical intervention with wide margins, checked by frozen sections during the surgery in almost 20 points followed by radiotherapy and chemotherapy. The interval between the first symptom and diagnosis in this case was almost 1.5 months which is shorter than the average interval reported in other cases (3.5 months).^13^

MFH of bone aggressively infiltrates adjacent tissues or between muscle fibers and this can be responsible for a high recurrence rate. Surgical excision with wide margins, even excision of clinically normal soft tissues, must be the procedure of choice.^6 , 17 , 18^ Frozen sections during surgery covering all margins of the lesion seem to play an important role in preventing local recurrence, as employed in this case in 20 points around the margins. Radiotherapy after surgery is usually indicated. MFH is often resistant to chemotherapy; however, use of vincristin, cyclophosphamid, dactiomyein, Adriamycin, cisplatin (cisdiamminedichlro platinum), tyrosine kinase inhibitors and dacarbazine (DTIC—Dimethyl Triazeno Imidazole Carboxamide) have been reported. ^1 , 19^

The management of malignant fibrous histiocytoma of bone should be based on an early histopathologic diagnosis and immuno-histochemical evaluations to perform radical surgery followed by radiotherapy and chemotherapy. Close follow-up and monitoring after treatment is also important because recurrence is a frequent problem and early metastasis to the lungs is common.
